# Identification of hub genes regulating the cell activity and function of adipose-derived stem cells under oxygen-glucose deprivation

**DOI:** 10.3389/fmolb.2022.1025690

**Published:** 2022-11-08

**Authors:** Zhenyu Yang, Wei Lu, Zuoliang Qi, Xiaonan Yang

**Affiliations:** Plastic Surgery Hospital (PSH), Chinese Academy of Medical Sciences and Peking Union Medical College, Beijing, China

**Keywords:** adipose-derived stem cells (ADSCs), oxygen-glucose deprivation (OGD), high-throughput sequencing, cell death and survival, cell activity modulation

## Abstract

While oxygen-glucose deprivation (OGD) has been widely utilized in many cell lines to mimic certain biological changes, it has yet to be validated in mesenchymal stem cells. We performed RNA sequencing on adipose-derived stem cells (ADSCs) under hypoxic and glucose-free conditions after 4 h and 8 h. A total of 335 common differentially expressed genes (DEGs) were identified in the two OGD groups compared with the normal control group, consisting of 292 upregulated and 43 downregulated genes. Gene Ontology and Kyoto Encyclopedia of Genes and Genomes (KEGG) enrichment analyses indicated that DEGs are mainly involved in metabolic processes, programmed cell death, and DNA-binding transcription activator activity. Protein‒protein interaction and hub gene analysis revealed various potential hub genes, in which response to oxygen levels, the IL-17-related biological function and the hypoxia-inducible factor 1 signaling pathway have been of vital importance. In summary, changes in transcription factor activity may play pivotal roles in oxygen-glucose deprivation. Through RNA sequencing, we have a deeper understanding of the changes in ADSCs after OGD treatment, providing more precise insight into predicting and regulating the stemness of ADSCs.

## Introduction

Hypoxia is a fundamental component of the microenvironment and is associated with cell proliferation, cell differentiation and cell death. Appropriate hypoxic preconditioning of ADSCs can boost cell proliferation (Efimenko et al., 2011), promote transformation to vascular smooth muscle cells (Lin et al., 2020), increase chondrogenesis, reduce osteogenesis, and enhance VEGF expression (Chen et al., 2016; Xu et al., 2020; Hwang et al., 2020). Changes in glucose levels can affect cell metabolism and function. Glucose deprivation is frequently explored in tumor cell models. For instance, the glucose withdrawal model, a common model in tumor research, demonstrates that the deprivation of glucose can induce multiple cell death modes, including ATF4-mediated apoptosis and ROS-mediated cell death, as well as increase the chance of KRAS mutations (Yun et al., 2009; Graham et al., 2012; Iurlaro et al., 2017). Various nontransformed cells exhibit different growth characteristics under glucose deprivation. The improved survival of mesenchymal stem cells under glucose deficiency was discovered to be the result of increased autophagy (Roa-Mansergas et al., 2018).

While appropriate glucose deprivation may be a potential treatment in cancer, concomitant hypoxia increases epithelialization and promotes cell invasion, suggesting that the simultaneous variation in oxygen and glucose may induce a more aggressive phenotype in tumor cells (Jo et al., 2020). When ADSCs are deprived of oxygen and glucose, some studies have demonstrated that ADSCs might regulate survival potential and stemness through autophagy (Li et al., 2017; Yang et al., 2021). In our previous study, a preexperiment was carried out, and we developed an OGD model to imitate the hypoxic and glucose-free environment *in vivo* (Yang et al., 2022). We also regulated RIP3, a key target in necroptosis, to relieve the effect of OGD treatment. However, there remains a lack of comprehensive network evaluation and standard quantitative references for the impact of the OGD environment on mesenchymal stem cells.

In this study, we performed RNA sequencing (RNA-seq) on ADSCs after different durations of OGD pretreatment to explore the functional impact of oxygen and glucose levels on ADSCs at the cellular level to obtain a more comprehensive understanding of possible changes in cellular biological activities during the early stage of transplantation.

## Materials and methods

### Cell preparation and OGD treatment

We harvested and identified ADSCs and built an OGD model as previously described (Yang et al., 2022). Briefly, type 1 collagenase was used to extract ADSCs from human adipose tissue. We cultured the cells with mesenchymal stem cell medium (MSCM 7501 ScienCell Research Laboratories, United States) at 37 °C in 5% CO2 until they reached 80–90% confluence. Cells at passages three to five were used for all the experiments in this study. To establish the OGD model, we replaced the medium with Glu-free DMEM (Gibco; Thermo Fisher Scientific, United States) after three washes with PBS. The dishes were then placed in an airtight chamber equipped with a vacuum air pump and an inflator (CelCulture Esco Micro Pte. Ltd., Singapore) and flushed with a gas mixture of 1% O_2_, 5% CO_2_, and 94% N_2_.

The cells were divided into three groups: (a) NC: normal control group cells were maintained in normal culture medium with 21% O_2_, 5% CO_2_, and 74% N_2_; (b) OGD-4 h: cells with OGD treatment for 4 h; (c) OGD-8 h: cells with OGD treatment for 8 h.

### Cell proliferation test and death detection

Cell Counting Kit-8 (CK18, Dojindo, Japan) was used to detect cell proliferation according to the manufacturer’s protocols. Briefly, 10,000 cultured cells were seeded in 96-well plates overnight and then cultured in glucose-free DMEM under 1% O_2_ or in MSCM in a normal incubator as a control. After 4 h and 8 h, 10 μl of CCK-8 reagent was added to each well and incubated at 37 °C for 2 h. The absorbance of each sample, which was proportional to the activity level of cell proliferation, was measured at a wavelength of 450 nm using a microplate reader. Each group was prepared in triplicate. The experiment was repeated three times.

Annexin V/7-AAD double staining was performed to analyze the state of the cells under different OGD treatment times. Briefly, three groups of cells were harvested, centrifuged, and resuspended in PBS at a concentration of 1 × 10/ml. The cell suspension was incubated with annexin V conjugated with FITC (556,420, BD Biosciences, United States) and 7-AAD (559925, BD Biosciences, United States) for 15 min in the dark and loaded on a flow cytometer.

### RNA isolation, quantification and qualification

Total RNA was isolated from the control, OGD-4 h, and OGD-8 h groups using the TRIzol method. The total amount and integrity of RNA were assessed using the RNANano 6000 Assay Kit of the Bioanalyzer 2100 system (Agilent Technologies, CA, United States). RNA purity was evaluated by calculating the A260/A280 ratio, which should be between 1.8 and 2.0.

### Library preparation and transcriptome sequencing

Total RNA was used as input material for the RNA sample preparations. Briefly, mRNA was purified from total RNA by using poly-T oligo-attached magnetic beads. First strand cDNA was synthesized using random hexamer primers and M-MuLV Reverse Transcriptase, while second strand cDNA synthesis was subsequently performed using DNA Polymerase I and dNTPs. After adenylation of the 3’ ends of DNA fragments, adaptors with hairpin loop structures were ligated to prepare for hybridization. To preferentially select cDNA fragments 370–420 bp in length, the library fragments were purified with an AMPure XP system (BeckmanCoulter, Beverly, United States). Then, PCR amplification was performed, the PCR product was purified by AMPure XP beads, and the library was finally obtained.

After the library was constructed and qualified by Qubit 2.0 Fluorometer, the different libraries were pooled according to the effective concentration and the target amount of data off the machine and then sequenced by the Illumina NovaSeq 6000 (Illumina, Inc., San Diego, CA, United States). The image data measured by the high-throughput sequencer are converted into sequence data (reads) by CASAVA base recognition. Clean data were obtained by removing reads containing adapters, reads containing N bases, and low-quality reads from the raw data. The index of the reference genome was built using HISAT2 (v2.0.5), and paired-end clean reads were aligned to the reference genome using HISAT2. FeatureCounts (v1.5.0-p3) was used to count the read numbers mapped to each gene.

### Identification of DEGs

Intergroup differences and intragroup sample duplications were evaluated *via* principal component analysis (PCA) (Yang et al., 2004). The R package DESeq2 was used to identify DEGs with a cutoff value of *p* value <0.01 and |log2-fold change| > 1 (Love et al., 2014). The original read count was standardized (normalization), and then the BH method was used for multiple hypothesis testing corrections. The visualization of DEGs was drawn *via* volcano plots, heatmap plots, and Venn diagrams by the ggplot2, pheatmap and VennDiagram packages in R language (Chen and Boutros, 2011; Wickham, 2011; Kolde and Kolde, 2015).

### Gene Ontology (GO) and Kyoto Encyclopedia of Genes and Genomes

The biological functions of the shared DEGs were explored with Gene Ontology and Kyoto Encyclopedia of Genes and Genomes (KEGG) enrichment analyses (Ashburner et al., 2000; Kanehisa and Goto, 2000). GO is the most extensive standardized gene function classification system, classifying the genes into three functional categories, including biological process (BP), cellular component (CC), and molecular function (MF), while KEGG is a visual systematic analysis database that summarizes cellular functions and metabolic pathways of genes. We performed GO and KEGG pathway analyses using the clusterProfiler package. Significant GO and KEGG pathways with the thresholds of q value <0.05 and *p* value < 0.05 were selected for further analysis. In addition, the ggplot2 and GOplot packages were used for cluster analysis. Networks were also generated in the Cytoscape software environment with the EnrichmentMap plugin using an uncorrected *p* value threshold of 0.005, an FDR cutoff of 0.1, and an overlap coefficient threshold of 0.1.

### Protein–protein interaction network analysis and module analysis

Protein–protein interaction (PPI) analysis was performed using an online database named Search Tool for the Retrieval of Interacting Genes (STRING). DEGs with a minimum required confidence score ≥0.7 were chosen to build a full network model, which was then visualized in Cytoscape software. In the network, each node corresponds to a protein, and each edge represents an interaction. MCODE, a molecular composite detection plugin in Cytoscape, was used to filter out hub genes from numerous candidates. The parameters were set as follows: Degree Cutoff = 2, Node Score Cutoff = 0.2, K-Core = 2, Max. Depth = 100. The most common and largest module was defined as having an MCODE score >4.

### Hub gene identification and bioinformatics analysis

Hub genes were screened by the combination of 12 prediction algorithms in the Cytoscape plugin cytoHubba (Chin et al., 2014). For each of the twelve algorithms (MCC, DMNC, MNC, Degree, EPC, BottleNeck, EcCentricity, Closeness, Radiality, Betweenness, Stress and Clustering Coefficient) in the cytoHubba plugin within Cytoscape, we identified the top 30 genes as hub genes. When a gene was identified as a hub gene in at least six algorithms, this gene was considered the final hub gene. The hub genes were analyzed *via* the clusterProfiler package.

### Statistical analysis

All quantitative results are presented as the means ± standard deviations (SD). Statistical comparisons were performed using one-way ANOVA in three independent experiments. GraphPad Prism version 9.0 software (GraphPad Inc., United States) was used for data analysis. Statistical significance was set at *p* < 0.05.

## Results

### Cell treatment

**FIGURE 1:**
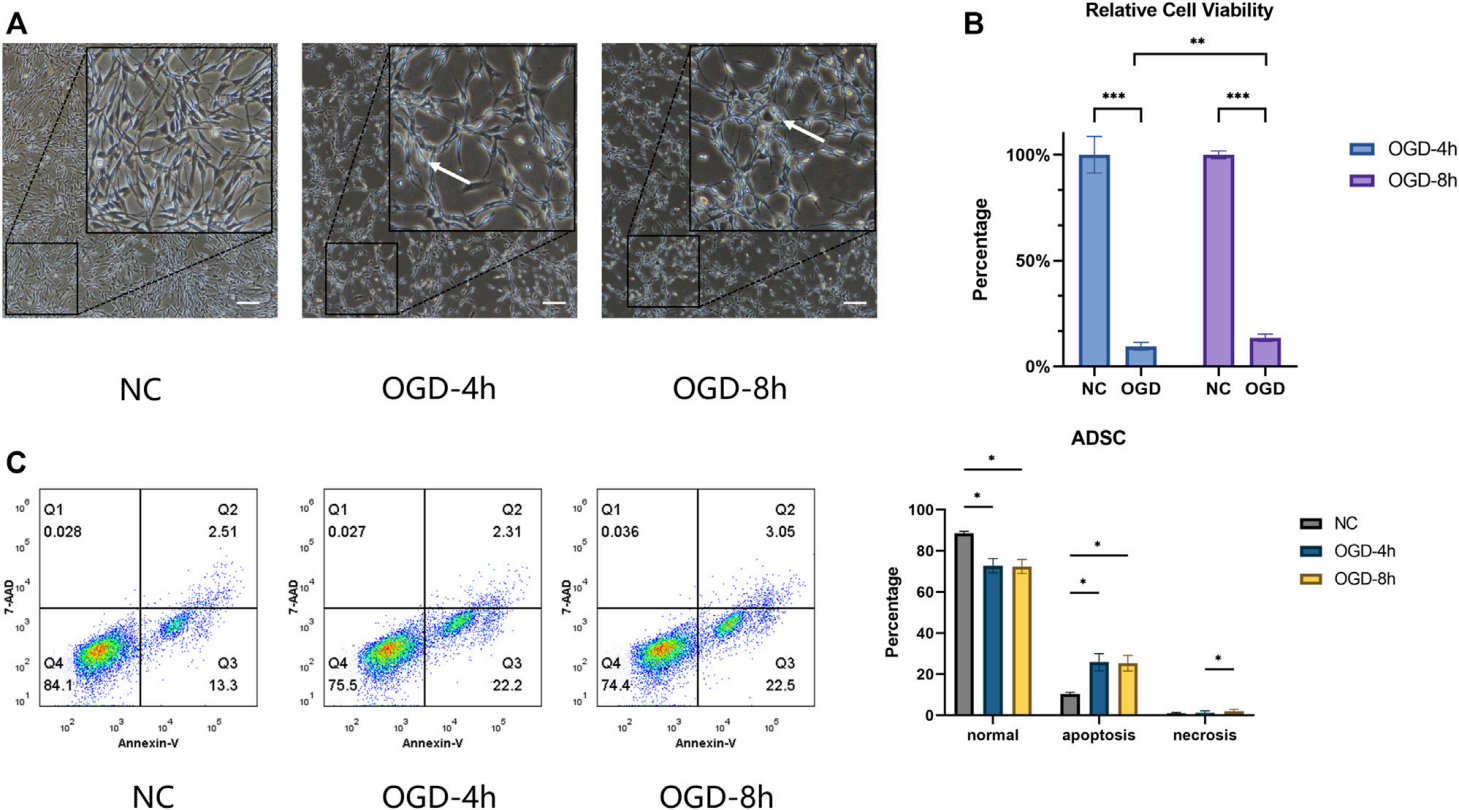
**(A)** Optical microscope morphology of ADSCs after normal culture and OGD treatment at different times. The round cells marked by arrows indicate that the cells are floating, suggesting that cell proliferation is affected, unable to adhere to the well normally, and concomitant cell death may occur. Scale bars = 200 µm. **(B)** ADSCs under OGD for 4 h and for 8 h represent relative cell viability of 9.52 ± 1.50% and 13.53 ± 1.76%, respectively. Significant differences were observed within the two subgroups compared with the control group and between the OGD-4 h and OGD-8 h groups. **(C)** The percentage of cells within the NC, OGD-4 h and OGD-8 h groups in the lower left (normal) quadrant was 88.57 ± 0.99%, 72.80 ± 3.46%, and 72.47 ± 3.44%, in the lower right (apoptosis) quadrant was 10.35 ± 0.83%, 25.93 ± 4.15%, and 25.40 ± 3.80%, and in the upper right (necrosis) quadrant was 1.04 ± 0.41%, 1.26 ± 0.91%, and 2.15 ± 0.80%, respectively. *p < 0.05, **p < 0.01, ***p < 0.001.

After being treated with OGD for 4 h or 8 h, some ADSCs were separated from the bottom of a culture dish, and the number of floating cells was measured (Figure 1A). OGD treatment suppressed cell viability, and a small increase in cell viability was observed at 8 h when compared with the OGD-4 h group (Figure 1B). The results of flow cytometry demonstrated that the cell survival rate of the NC group was higher than that of the OGD-4 h and OGD-8 h groups, the apoptosis rate demonstrated the opposite trend, and the number of annexin V single-stained cells in the third quadrant increased significantly, suggesting that OGD would induce more apoptosis in ADSCs, but there was no significant difference between the OGD-4 h group and the OGD-8 h group. In addition, no significant difference regarding the necrosis rate was observed between the NC and OGD-treated groups (Figure 1C).

### Sequencing data preprocessing

To imitate the environment *in vivo*, we cultured ADSCs under hypoxic and glucose-free conditions and performed high-throughput sequencing at three different time intervals (0, 4, and 8 h) to further identify the underlying mechanism in the pathophysiological process. After filtering the original data, checking the sequencing error rate, and verifying the distribution of GC content, clean reads for follow-up analysis were obtained. The 4 h samples OGD4h_1, OGD4h_2 and OGD4 h _3 generated 42.36, 40.77 and 45.51 million clean reads, while the 8 h samples OGD8h_1, OGD8h_2 and OGD8 h _3 generated 52.28, 45.78 and 46.14 million clean reads, respectively. Normal control samples NC_1, NC_2, and NC_3 generated 42.83, 42.97, and 39.58 million clean reads, respectively (Supplementary Table S1A). Quality-controlled clean reads were compared to the human reference genome. The results of the comparison of each sample in this project with the human reference genome are presented in Supplementary Table S1B. The total mapped reads for OGD4h_1, OGD4 h _2 and OGD4h_3 were 95.23, 96.13% and 95.51, respectively, whereas those for OGD8h_1, OGD8 h _2, OGD8h_3, NC_1, NC_2 and NC_3 were 92.25, 94.9, 96.07, 93.5, 94.31 and 95.73%, respectively.

### Identification of differentially expressed genes in the OGD model

**FIGURE 2:**
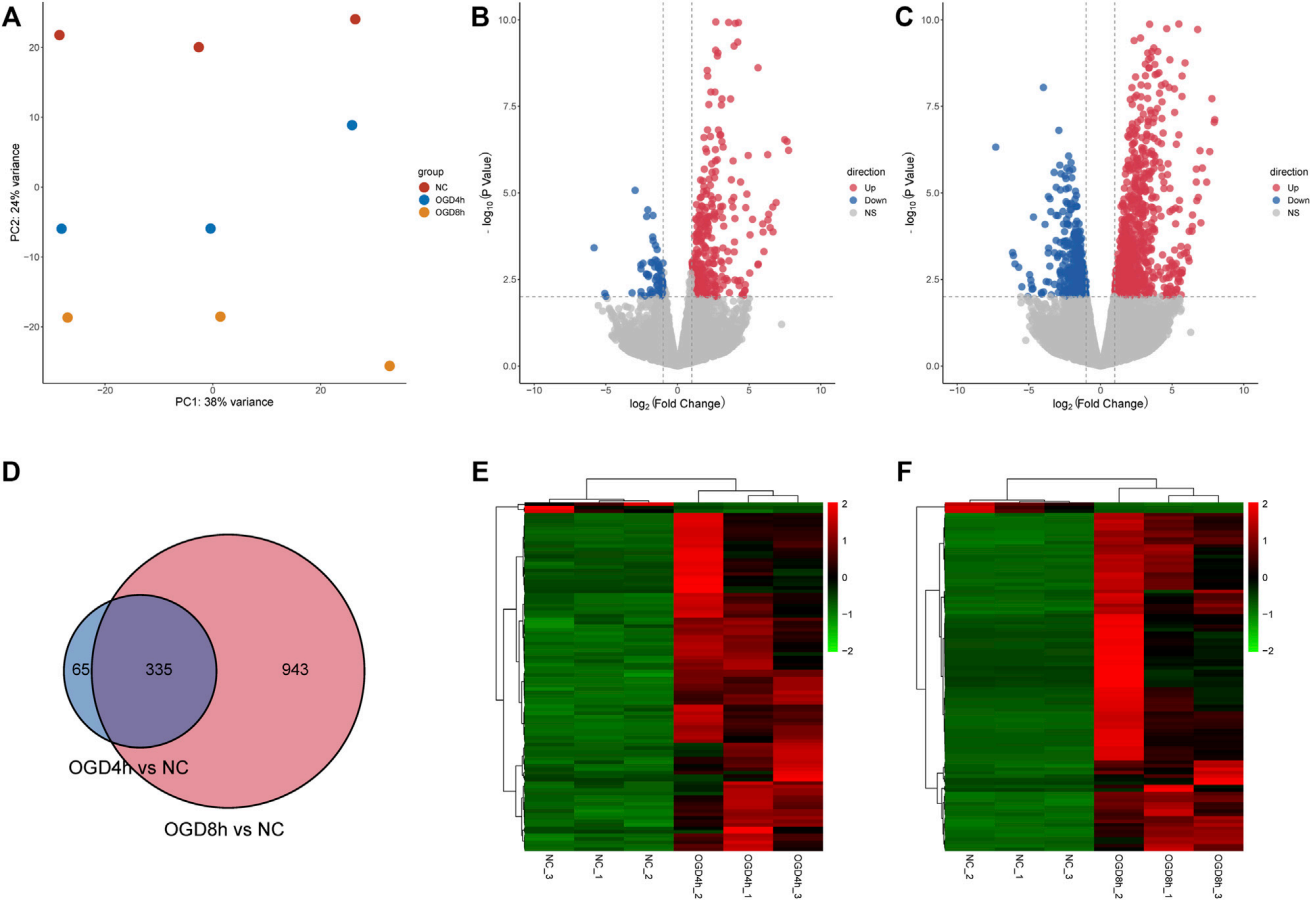
**(A)** Principal component analysis diagram showing that samples of different groups were significantly different, and samples within the same group were relatively uniform. **(B)** Volcano plot showing the distribution of log2-fold change and–log10 *p* value of all quantified proteins in the 4 h model. Blue circles: *p* < 0.01, log2-fold change <−1; red circles: *p* < 0.01, log2-fold change >1. **(C)** Volcano plot showing the distribution of log2-fold change and–log10 *p* value of all quantified proteins in the 8 h model. Blue circles: *p* < 0.01, log2-fold change <−1; red circles: *p* < 0.01, log2-fold change >1. **(D)** The Venn diagram shows DEGs identified at two time points compared to the normal control group. A total of 335 DEGs overlapped between the 4 h group and the 8 h group. **(E)** Heatmaps showing gene expression values for the differentially expressed genes (DEGs) at 4 h compared with the normal control group. **(F)** Heatmaps showing gene expression values for the differentially expressed genes (DEGs) at 8 h compared with the normal control group.

Data cross-comparability was evaluated *via* PCA to confirm biological variability between different samples. All samples were grouped separately (Figure 2A), and the first principal component axis (PC1) accounted for 38% of the explained variance in the data, indicating globally distinct expression profiles.

A total of 400 DEGs were identified after screening at |log2-fold change|>2 and *p* value < 0.01 at 4 h, while 335 DEGs were upregulated and 65 DEGs were downregulated. The volcano plot (Figure 2B) and heatmap (Figure 2E) of the DEGs indicated that these genes could easily distinguish the OGD4 h group from the NC group, suggesting that they may play a critical role in the development of oxygen-glucose deprivation. At 8 h, 1,278 DEGs were identified compared to the normal control group (Figures 2C,F). Of these transcripts, 884 were upregulated, and 394 were downregulated. Remarkably, a total of 335 shared DEGs were identified in the OGD8 h compared to control samples (Figure 2D), including 292 upregulated and 43 downregulated genes.

### Functional enrichment analysis of differentially expressed genes

**FIGURE 3:**
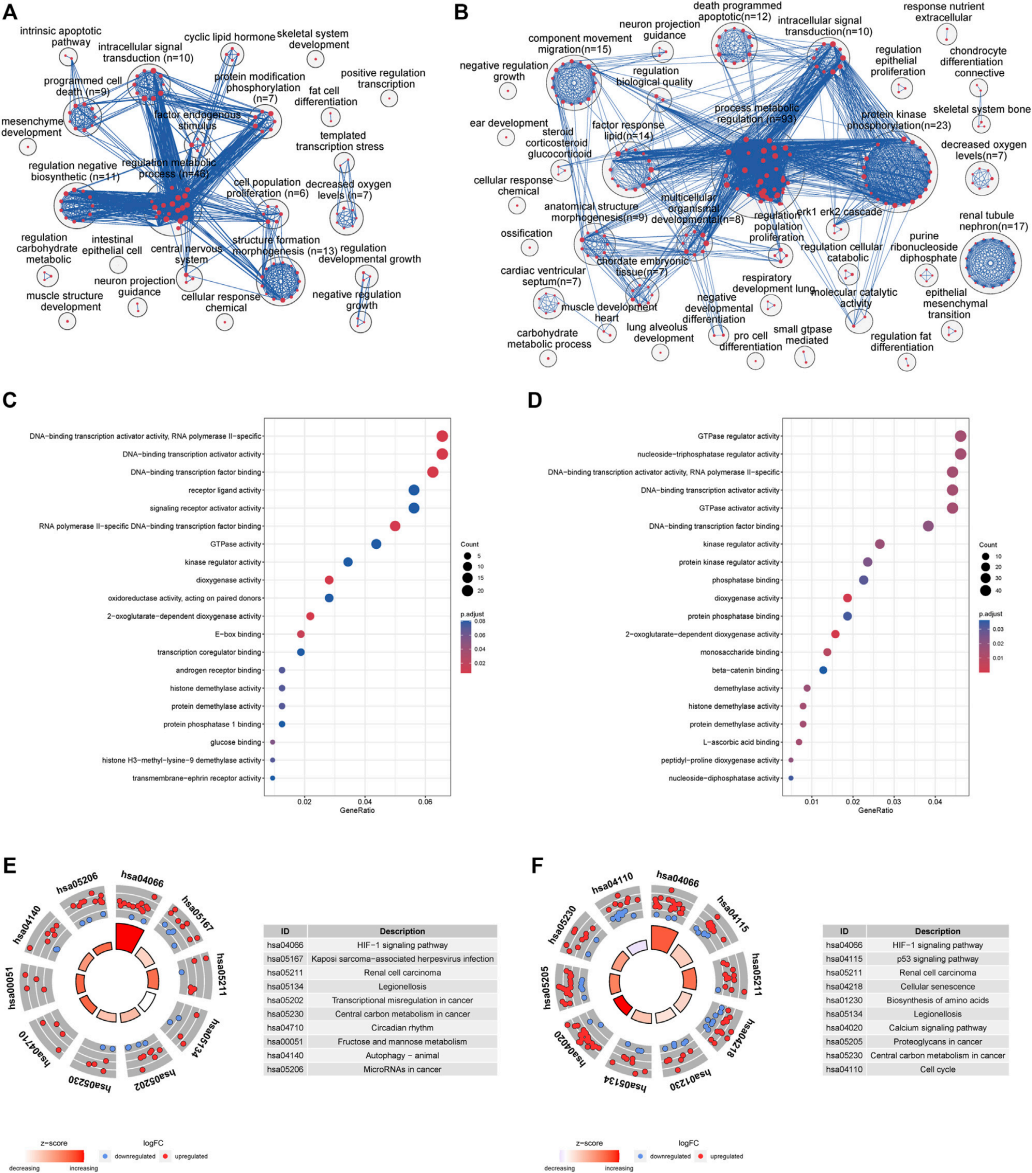
**(A)** Enrichment analysis identified over 500 significantly enriched biological processes that were clustered using EnrichmentMap and AutoAnnotate in Cytoscape to identify the key biological processes involved in oxygen-glucose deprivation at 4 h. Nodes represent individual GO terms, with size relating to the number of genes in each term and the color indicating enrichment significance. Edges represent connections between nodes that share genes. **(B)** Enrichment analysis at 8 h **(C)** GO enrichment analyses of the top 20 most significantly enriched molecular functions of the shared DEGs at 4 h **(D)** GO enrichment analyses of the top 20 most significantly enriched molecular functions of the shared DEGs at 8 h **(E)** KEGG enrichment analyses of the top 10 most significant pathways at 4 h; the inner ring is a bar plot where the bar height indicates the significance of the term (−log10 *p* value), and the color indicates the z score. The outer ring displays scatterplots of the expression levels (log2-fold change) for the genes in each term. **(F)** KEGG enrichment analyses of the top 10 most significant pathways at 8 h.

Enrichment analysis identified over 300 significantly enriched biological processes (Supplementary Table S2). To facilitate a more straightforward interpretation of functional enrichment, a network of biological process terms was created using EnrichmentMap. DEGs were significantly enriched in “regulation of metabolic process,” “programmed cell death” and “cell proliferation” at 4 h (Figure 3A), while DEGs were mainly involved in “regulation of metabolic process,” “protein kinases mediate phosphorylation,” “apoptosis” and “intracellular signal transduction” at 8 h (Figure 3B). In addition, molecular function analysis showed that DEGs were mainly associated with “DNA−binding transcription activator activity” and “dioxygenase activity” at 4 h (Figure 3C), while DEGs were involved in “GTPase regulator activity,” “nucleoside−triphosphatase regulator activity” and “DNA−binding transcription activator activity”at 8 h (Figure 3D).

KEGG pathway enrichment analysis showed that the DEGs were enriched in 15 signaling pathways at 4 h (Figure 3E, Supplementary Table S3A), including the HIF-1 signaling pathway, legionellosis, fructose and mannose metabolism, autophagy and microRNAs. Meanwhile, the significantly enriched KEGG pathways of shared DEGs at 8 h included the HIF-1 signaling pathway, p53 signaling pathway, cellular senescence, and biosynthesis of amino acids (Figure 3F, Supplementary Table S3B).

### Protein interaction network construction of DEGs and module analysis

**FIGURE 4:**
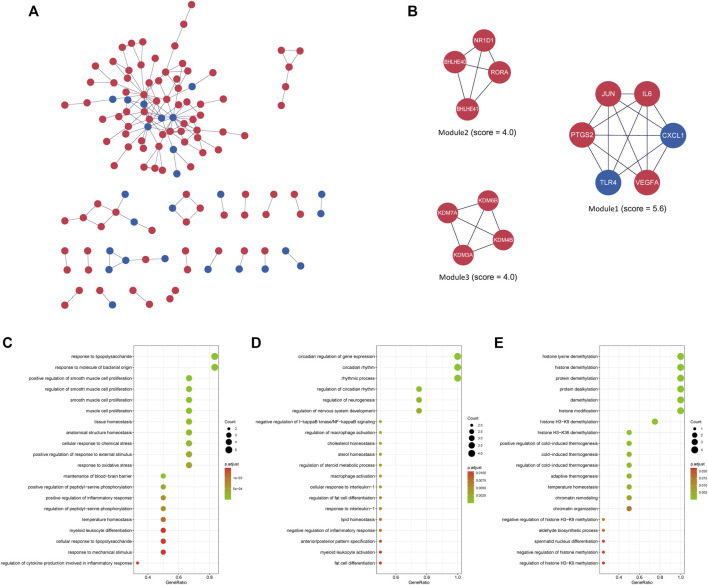
**(A)** The interaction network between proteins coded by DEGs comprised 125 nodes and 157 edges in the 4 h model. The red and blue circle nodes represent upregulated and downregulated DEGs, respectively. **(B)** Three cluster modules extracted by MCODE. **(C)** GO enrichment analysis of Module 1. **(D)** GO enrichment analysis of Module 2. **(E)** GO enrichment analysis of Module 3.

**FIGURE 5:**
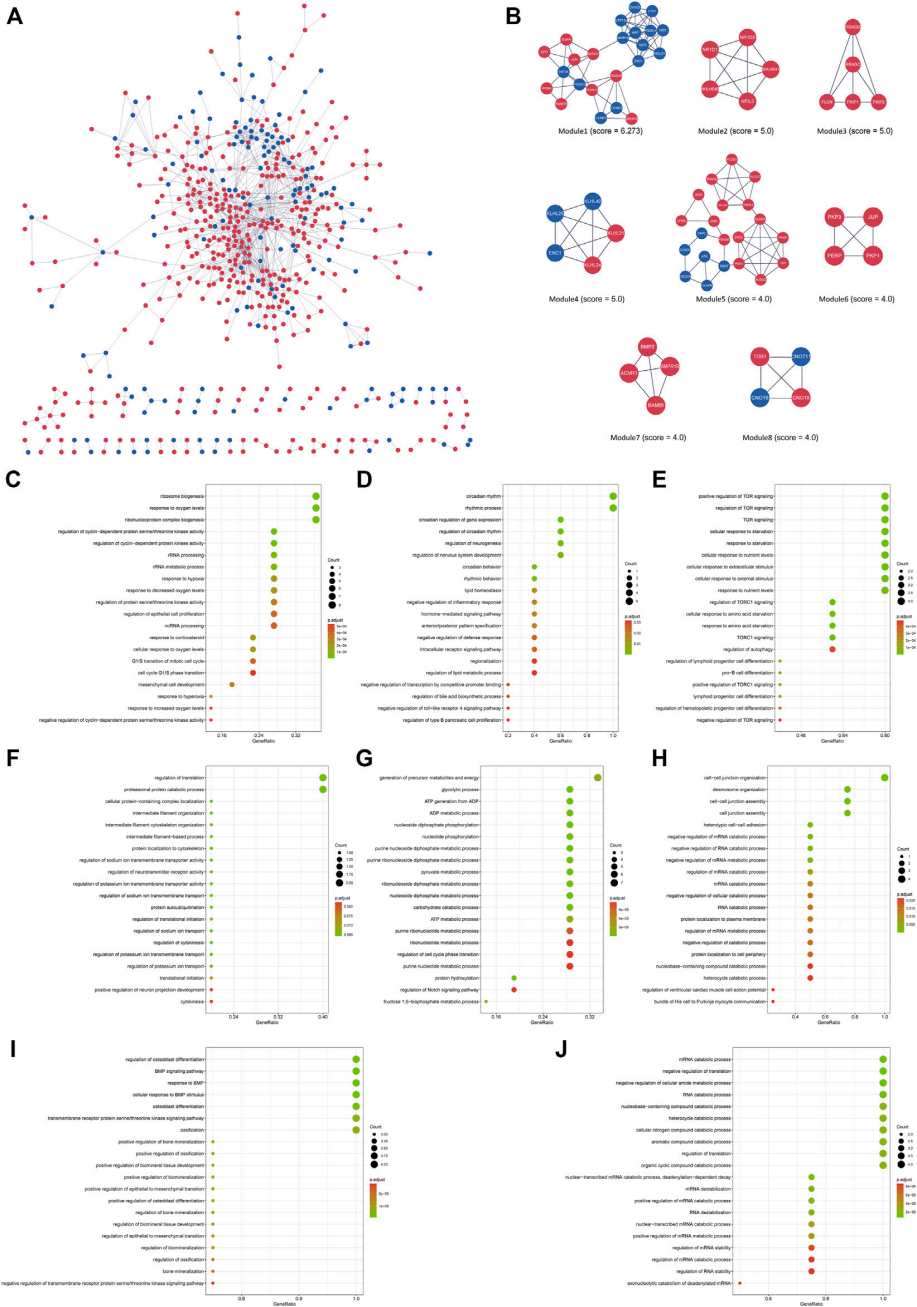
**(A)** The interaction network between proteins coded by DEGs comprised 563 nodes and 1,050 edges in the 8 h model. The red and blue circle nodes represent upregulated and downregulated DEGs, respectively. **(B)** Eight cluster modules extracted by MCODE. **(C)** GO enrichment analysis of Module 1. **(D)** GO enrichment analysis of Module 2. **(E)** GO enrichment analysis of Module 3. **(F)** GO enrichment analysis of Module 4. **(G)** GO enrichment analysis of Module 5. **(H)** GO enrichment analysis of Module 6. **(I)** GO enrichment analysis of Module 7. **(J)** GO enrichment analysis of Module 8. L.

The interactions between the proteins expressed from differentially expressed genes at 4 h of the oxygen-glucose deprivation model consisted of 125 nodes and 157 edges in Figure 4A, while the PPI networks at 8 h after oxygen-glucose deprivation are presented in Figure 5A, with 563 nodes and 1,050 edges.

In the MCODE analysis of the 4 h model, a total of three modules with MCODE scores >4 were obtained (Figure 4B). Module 1, with the highest score of 5.6, comprised six genes, including JUN, IL6, CXCL1, VEGFA, TLR4 and PTGS2 (COX2), all of which were defined as the main hub nodes by cytoHubba in the PPI network, indicating that module 1 may be the main functional module. Module 2, comprised of RORΑ, NR1D1, BHLHE40 and BHLHE41, was scored four in MCODE. Module 3 consisted of four genes, including KDM3A, KDM4B, KDM6B and KDM7A. The score of module 3 was 4. RORΑ, KDM3A, KDM4B, KDM6B and KDM7A were identified as key genes by cytoHubba in modules 2 and 3. To further investigate the biological functions of the modules, GO and KEGG analyses were performed (Figures 4C–E, Supplementary Table S4A). Module 1 was enriched in 813 GO terms, including “response to lipopolysaccharide,” “positive regulation of smooth muscle cell proliferation,” and “response to molecule of bacterial origin” (Figure 4C), while module 2 was enriched in 166 GO terms, including “circadian regulation of gene expression,” “circadian rhythm,” and “rhythmic process” (Figure 4D). Module 3 was associated with demethylation, suggesting that demethylation might play an important role in oxygen-glucose deprivation in adipose-derived stem cells (Figure 4E). Supplementary Table S4A shows the KEGG analysis results of the three modules.

In the 8 h model, eight key modules were identified based on the Cytoscape plugin MCODE (Figure 5B). Herein, EGFR, NOTCH1, JUN, HIF1A, FOXO1, RORΑ, CDKN1A (p21) and CDKN1B (p27) were predicted to be hub genes in the cytoHubba plugin, suggesting that they may play a critical role in the development of oxygen-glucose deprivation in adipose-derived stem cells. GO and KEGG enrichment analyses of these modules were conducted (Figures 5C–J, Supplementary Table S4B). For example, the genes of Module 1 were enriched in the cell cycle, while the genes of Module 2 were associated with circadian rhythm. Modules 3–8 were enriched in TOR signaling, regulation of translation, generation of precursor metabolites and energy, cell−cell junction organization, osteoblast differentiation and mRNA catabolic process, respectively.

### Enrichment analysis of hub genes in cytoHubba

**FIGURE 6:**
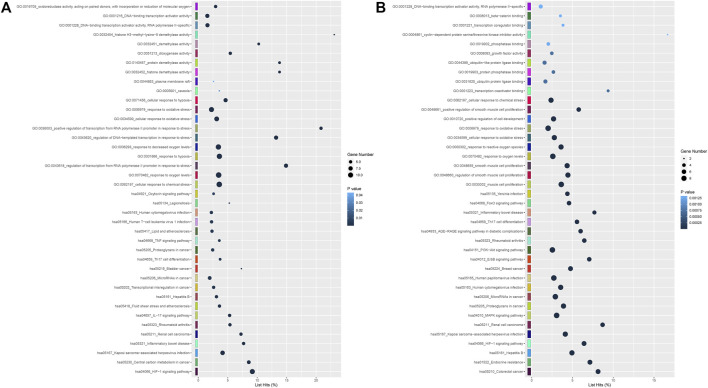
**(A)** GO including biological process, cellular components and molecular function analysis, and KEGG enrichment analysis of hub differentially expressed at 4 h after genes oxygen-glucose deprivation. **(B)** GO and KEGG enrichment analyses of differentially expressed hub genes at 8 h.

As shown in Supplementary Table S5A, a total of 30 genes, which were predicted by more than six cytoHubba algorithms, were identified as hub genes in the 4 h model, including ATF3, CA9, CBX8, CDKN1A, CDK5R1, CXCL1, DDIT3, HIF1A, HK2, HMOX1, IL6, JUN, KDM3A, KDM4B, KDM6B, KDM7A, LEP, MAF, MAPK7, MEF2A, MYC, PDK1, PFKFB3, PTGS2, RORΑ, SLC16A3, SLC2A1, SLC2A3, TLR4 and VEGFA, with HMOX1, IL6, KDM3A and TLR4 predicted by twelve algorithms. Hub genes were enriched in many GO and KEGG terms, including “HIF-1 signaling pathway,” “oxidoreductase activity, acting on paired donors, with incorporation or reduction of molecular oxygen” and “DNA−binding transcription activator activity”. (Figure 6A).

In the 8 h model, 20 genes, including CDKN1A (p21), CDKN1B, CRKL, CYCS (CytC), EGFR, FOS, FOXO1, HIF1A, IL6, JUN, MYC (C-Myc), NGF, NOTCH1, NOTCH3, PDGFRB, PXN, RORΑ, TGFB1, TLR4, VEGFA and WDR12, were identified as hub genes in Supplementary Table S5B. None of the hub genes were predicted by 12 algorithms in the 8 h model. The results from GO and KEGG pathway enrichment analysis demonstrated that hub genes were significantly involved in “FoxO signaling pathway,” “PI3K-Akt signaling pathway,” “beta−catenin binding” and “DNA−binding transcription activator activity, RNA polymerase II−specific” (Figure 6B).

## Discussion

Most of the studies exploring oxidative stress and glycometabolism investigated the proliferation and death of ADSCs exposed to hypoxia without glucose level modification (von Heimburg et al., 2005; Xu et al., 2016, 2020bOng et al., 2017; Suszynski et al., 2019). Given the microenvironment of the cells administered *in vivo*, we speculate that hypoxia and a low glucose level were associated with stemness maintenance and contributed to the decreased cell number and increased apoptosis. Therefore, we searched for genes with potential roles in ADSCs under oxygen-glucose deprivation. Three time points (0, 4, and 8 h) in the oxygen-glucose deprivation phase were chosen, and the high-throughput sequencing data identified hub genes guiding cell proliferation and death.

At two time points of oxygen-glucose deprivation, gene expression analyses revealed a higher number of differentially expressed genes at 8 h. A total of 335 DEGs overlapped between the 4 h group and the 8 h group (Figure 2D), including 292 upregulated and 43 downregulated genes. To better understand the interactions of DEGs, we further performed GO, KEGG pathway, and PPI network analyses. Biological process analysis indicated that DEGs mainly participated in metabolic regulation, protein kinases mediate phosphorylation, cell proliferation and programmed cell death (apoptosis) (Figures 3A,B), consistent with our flow cytometry results. Cellular component and molecular function analyses demonstrated that DNA-binding transcription activator activity, E-box binding, transcription coregulator binding, histone demethylase activity and histone H3-methyl-lysine-9 demethylase activity (Figures 3C,D), indicating that transcription factors were crucial in the cytopathological process of hypoxia and hypoglycemia in ADSCs. Furthermore, the enriched KEGG pathways of DEGs at 4 h included the HIF-1 signaling pathway, legionellosis, transcriptional misregulation, central carbon metabolism, circadian rhythm, fructose and mannose metabolism and autophagy (Figure 3E), and those at 8 h included the HIF-1 signaling pathway, p53 signaling pathway, cellular senescence, biosynthesis of amino acids, legionellosis, calcium signaling pathway, central carbon metabolism and cell cycle (Figure 3F). Therefore, all these pathways could contribute to the cytopathological process of oxygen-glucose deprivation in ADSCs. Remarkably, HIF overexpression and the HIF pathway have been considered to be independent prognostic factors in many carcinomas (Zhong et al., 1999; McGettrick and O’Neill, 2020), although limited data are available in hypoxic and hypoglycemic human adipose-derived stem cells. Our study concluded that the HIF-1 pathway, characterized by nuclear overexpression of HIF-1a, correlated with stemness maintenance, cell proliferation and cell death (Figures 3E,F). Moreover, IL-6, TLR4, VEGFA, PDK1, CDKN1A, CDKN1B and HMOX1, all of which are part of the HIF-1 signaling pathway, were identified as hub genes or key modules (Supplementary Figure S1). The IL-17 signaling pathway, including MAPK, IL-6, PTGS2 and CXCL1, was identified as a hub gene (Supplementary Figure S2), and the FoxO signaling pathway, including TGFB, IL-6, FOXO1, EGFR, CDKN1A, CDKN1B and MYC (Supplementary Figure S3), was another pathway that contributed greatly to oxygen- and glucose-deprived ADSCs. Histone lysine demethylases (KDM family), consisting of eight subfamilies KDM1-8, are enzymes that remove methylation marks on lysines in nucleosome histone tails, indicating that epigenetic modifications are critical in the cytopathological process of hypoxia and hypoglycemia in ADSCs(Zhang et al., 2012; Sterling et al., 2020). Remarkably, the HIF-1, FoxO and IL-17 pathways are all nuclear receptor signaling pathways that can be modulated through the action of numerous growth factor and cytokine signaling cascades, confirming that transcription factors may play a crucial role in the study. In the KEGG pathway, hsa05202 refers to transcriptional misregulation in cancer, and we found that most of the genes in the pathway were marked red, indicating that most transcription factors were significantly upregulated or downregulated in oxygen- and glucose-deprived ADSCs (Supplementary Figure S4). DNA-binding transcription activator activity ranked high in GO analysis (Figures 3C,D), characterized by significantly adjusted *p* values. These findings suggest the importance of transcription factors in the OGD model.

In the identification of key modules and hub genes, JUN, IL6, CXCL1, VEGFA, TLR4, PTGS2 (COX2), RORΑ, KDM3A, KDM4B, KDM6B, KDM7A, EGFR, NOTCH1, JUN, HIF1A, FOXO1, RORΑ, CDKN1A (p21), and CDKN1B (p27) were characterized with the highest score (Supplementary Table S5), and they also belonged to crucial modules of the PPI network (Figures 4A, 5A). Most of them were part of the IL-17 signaling pathway, HIF1 signaling pathway, and FoxO signaling pathway, including IL6, CXCL1, VEGFA, TLR4, PTGS2, HIF1A, FOXO1, CDKN1A and CDKN1B. Retinoic acid receptor-related orphan receptor alpha (RORα) is a member of the nuclear receptor superfamily of ligand-regulated transcription factors that link inflammatory and metabolic signaling pathways (Jetten, 2009). A study by Rebeca Ortega found that activation of RORα might promote inflammation during adipose tissue expansion, resulting in metabolic dysfunction (Ortega et al., 2021). Thus, upregulation of RORα might play a critical role in the cytopathological process of hypoxia and hypoglycemia in ADSCs. The Kelch-like gene family (KLHL), containing KLHL21, KLHL24, KLHL25, and KLHL42 in the hub module analysis at 8 h (Figure 5B), is involved in the ubiquitination process, but its specific roles have not yet been elucidated (Dhanoa et al., 2013). The mechanism underlying the KLHL family suggests that the ubiquitination process may play a critical role in the hypoxic and hypoglycemic environment.

In general, through bioinformatic research methods, we identified genes differentially expressed in the cytopathological process of hypoxia and hypoglycemia compared with the normal group and explored their potential function and relevant pathways. Changes in transcription factor activity and the HIF-1 pathway may play pivotal roles, and further experiments are required to further clarify the effect of these hub genes.

## Data Availability

The datasets presented in this study can be found in online repositories. The names of the repository/repositories and accession number(s) can be found below: Sequence Read Archive with a BioProject accession number PRJNA874355.

## Supplementary material

SUPPLEMENTARY FIGURE S1 Gene expression changes in the HIF-1 signalling pathway. Representation of the differentially expressed genes that were enriched in the HIF-1 signalling pathway (KEGG: hsa04066). Red boxes indicate the differentially expressed genes.

SUPPLEMENTARY FIGURE S2 Gene expression changes in the IL-17 signalling pathway. Representation of the differentially expressed genes that were enriched in the IL-17 signalling pathway (KEGG: hsa04657). Red boxes indicate the differentially expressed genes.

SUPPLEMENTARY FIGURE S3 Gene expression changes in the FOXO signalling pathway. Representation of the differentially expressed genes that were enriched in the FOXO signalling pathway (KEGG: hsa04068). Red boxes indicate the differentially expressed genes.

SUPPLEMENTARY FIGURE S4 Gene expression changes in transcriptional regulation. Representation of the differentially expressed genes that were enriched in the transcriptional regulation (KEGG: hsa05202). Red boxes indicate the differentially expressed genes.

## Abbreviations

PCA, principal component analysis; DEG, differentially expressed gene; FDR, False Discovery Rate; GO, Gene Ontology; KEGG, Kyoto Encyclopedia of Genes and Genomes; BP, biological process; CC, cellular component; MF, molecular function; PPI, protein–protein interaction; OGD, oxygen-glucose deprivation.
