# Supplementary figures and images for: Identification of hub genes regulating the cell activity and function of adipose-derived stem cells under oxygen-glucose deprivation
Source: Front Mol Biosci. 2022 Nov 8;9:1025690. doi: 10.3389/fmolb.2022.1025690 (PMC9679370; doi:10.3389/fmolb.2022.1025690)

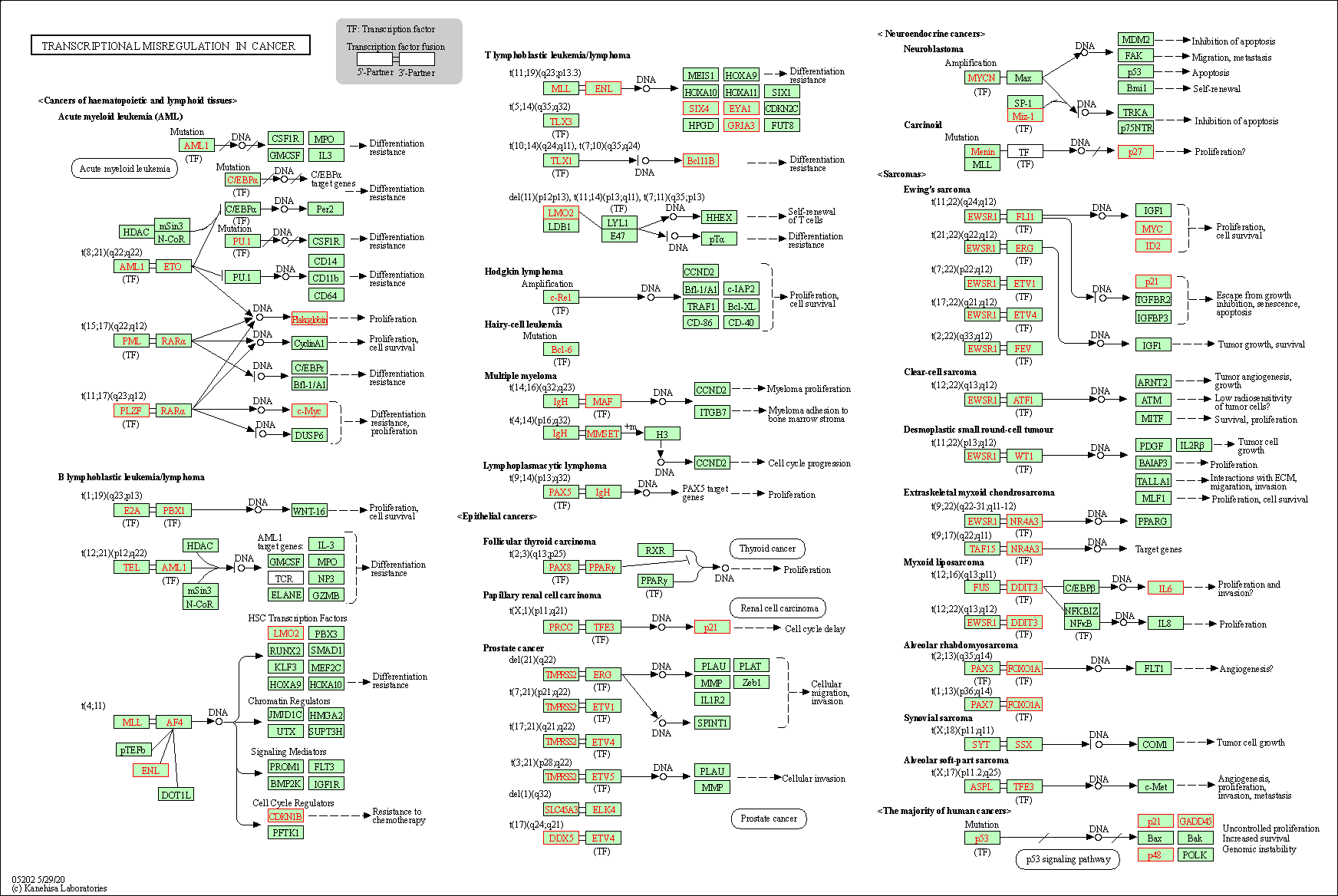

Supplement: Supplementary file 3 [file Image4.PNG]

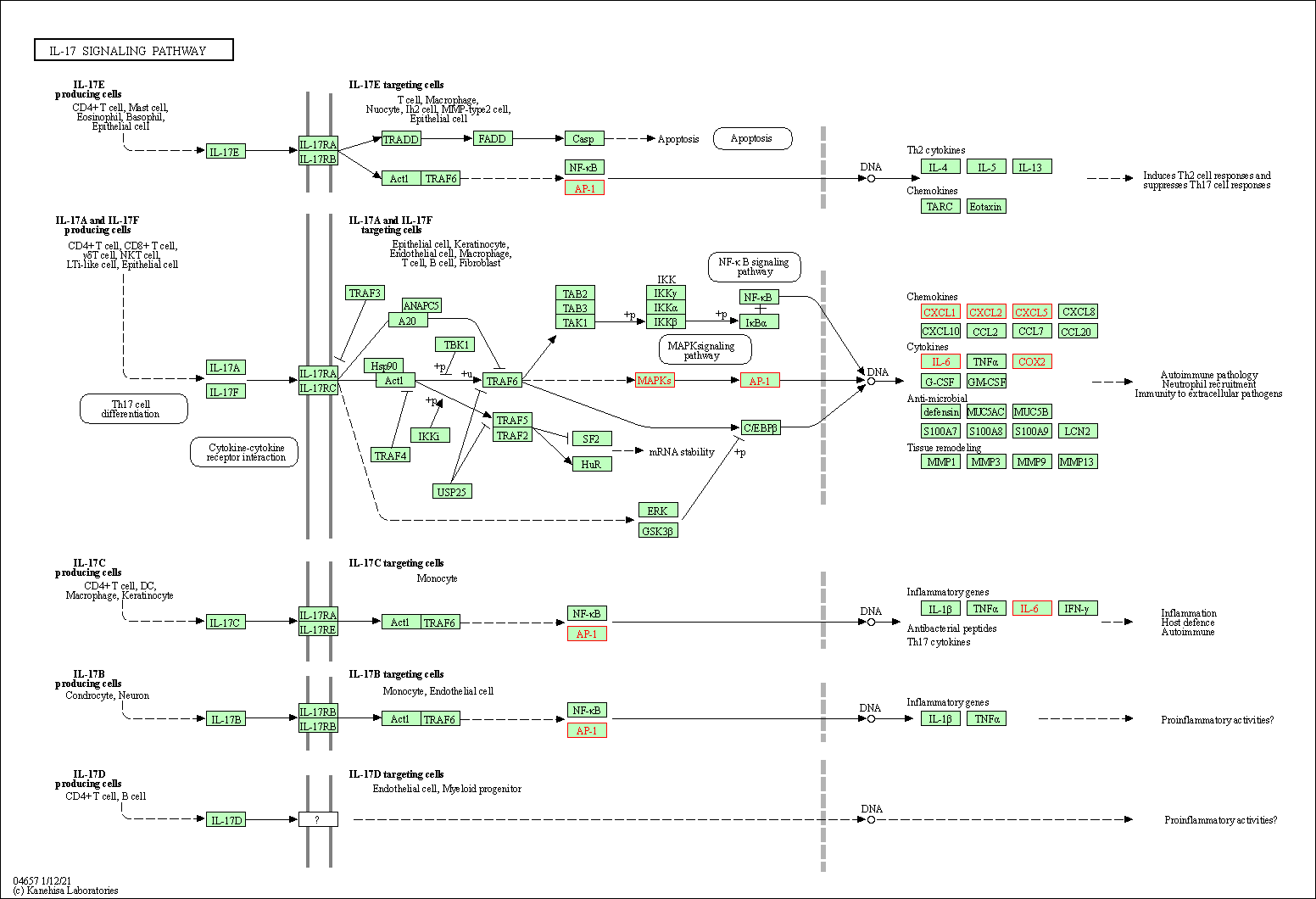

Supplement: Supplementary file 5 [file Image2.PNG]

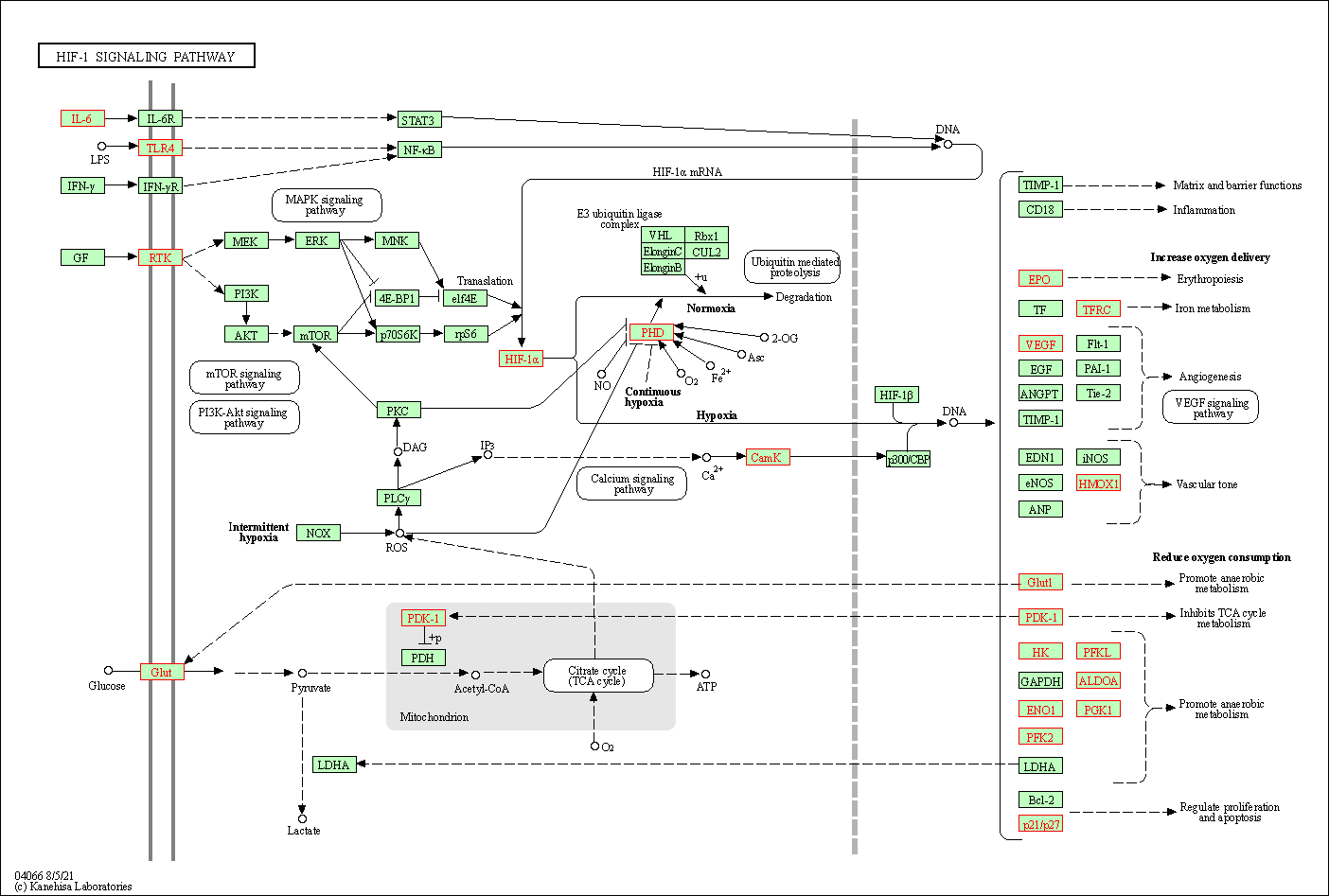

Supplement: Supplementary file 7 [file Image1.PNG]

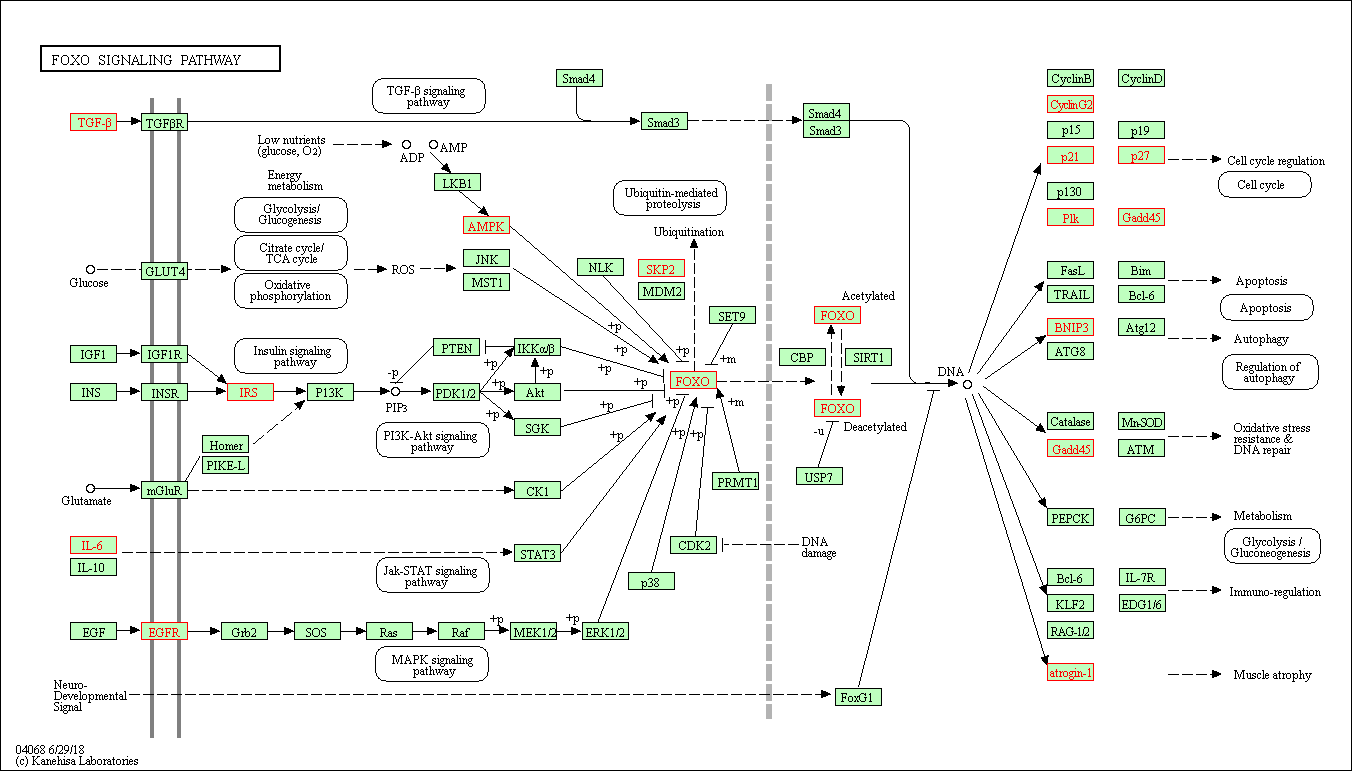

Supplement: Supplementary file 9 [file Image3.PNG]
