# Supplementary material for: Identification of hub genes regulating the cell activity and function of adipose-derived stem cells under oxygen-glucose deprivation
Source: Front Mol Biosci. 2022 Nov 8;9:1025690. doi: 10.3389/fmolb.2022.1025690 (PMC9679370; doi:10.3389/fmolb.2022.1025690)
Supplement: Supplementary file 10 [file DataSheet1.docx]

Figure S1. Gene expression changes in the HIF-1 signalling pathway. Representation of the differentially expressed genes that were enriched in the HIF-1 signalling pathway (KEGG: hsa04066). Red boxes indicate the differentially expressed genes.

Figure S2. Gene expression changes in the IL-17 signalling pathway. Representation of the differentially expressed genes that were enriched in the IL-17 signalling pathway (KEGG: hsa04657). Red boxes indicate the differentially expressed genes.

Figure S3. Gene expression changes in the FOXO signalling pathway. Representation of the differentially expressed genes that were enriched in the FOXO signalling pathway (KEGG: hsa04068). Red boxes indicate the differentially expressed genes.

Figure S4. Gene expression changes in transcriptional regulation. Representation of the differentially expressed genes that were enriched in the transcriptional regulation (KEGG: hsa05202). Red boxes indicate the differentially expressed genes.
